# Development and validation of a predictive model for 30-day mortality in patients with severe community-acquired pneumonia in intensive care units

**DOI:** 10.3389/fmed.2023.1295423

**Published:** 2024-01-08

**Authors:** Yu Zhang, Yuanyuan Peng, Wang Zhang, Wei Deng

**Affiliations:** ^1^Department of Infection Control, Chongqing Mental Health Center, Chongqing, China; ^2^Department of Critical Care Medicine, Chongqing General Hospital, Chongqing, China; ^3^Third Psychogeriatric Ward, Chongqing Mental Health Center, Chongqing, China; ^4^Department of Nursing, Chongqing Mental Health Center, Chongqing, China

**Keywords:** nomogram, predictive model, severe community-acquired pneumonia, intensive care units, 30-day mortality

## Abstract

**Background:**

Based on the high prevalence and fatality rates associated with severe community-acquired pneumonia (SCAP), this study endeavored to construct an innovative nomogram for early identification of individuals at high risk of all-cause death within a 30-day period among SCAP patients receiving intensive care units (ICU) treatment.

**Methods:**

In this single-center, retrospective study, 718 SCAP patients were screened from the Medical Information Mart for Intensive Care IV (MIMIC-IV) database for the development of a predictive model. A total of 97 patients eligible for inclusion were included from Chongqing General Hospital, China between January 2020 and July 2023 for external validation. Clinical data and short-term prognosis were collected. Risk factors were determined using the least absolute shrinkage and selection operator (LASSO) and multiple logistic regression analysis. The model’s performance was evaluated through area under the curve (AUC), calibration curve, and decision curve analysis (DCA).

**Results:**

Eight risk predictors, including age, presence of malignant cancer, heart rate, mean arterial pressure, albumin, blood urea nitrogen, prothrombin time, and lactate levels were adopted in a nomogram. The nomogram exhibited high predictive accuracy, with an AUC of 0.803 (95% CI: 0.756–0.845) in the training set, 0.756 (95% CI: 0.693–0.816) in the internal validation set, 0.778 (95% CI: 0.594–0.893) in the external validation set concerning 30-day mortality. Meanwhile, the nomogram demonstrated effective calibration through well-fitted calibration curves. DCA confirmed the clinical application value of the nomogram.

**Conclusion:**

This simple and reliable nomogram can help physicians assess the short-term prognosis of patients with SCAP quickly and effectively, and could potentially be adopted widely in clinical settings after more external validations.

## 1 Introduction

Community-acquired pneumonia (CAP) is an acute respiratory infection disease acquired in the community setting and can be caused by a large variety of microorganisms including bacteria, respiratory viruses, and fungi. Additionally, it presents as a public health concern linked to significant morbidity, mortality, and economic burden (1). A study conducted in the USA showed that the annual incidence was 649 patients hospitalized with CAP per 100,000 adults (2). Among the hospitalized CAP patients, 21% of them required intensive care unit (ICU) admission (3). Severe community-acquired pneumonia (SCAP), an advanced form of CAP, is one of the leading causes of death in ICU (4). It’s characterized by a systemic inflammatory response and often results in multiple organ dysfunction syndrome (MODS). While the prognosis of severe pneumonia has somewhat improved due to the escalation of antibiotics, as well as the use of mechanical ventilation and glucocorticoids, SCAP still exhibits a high hospital mortality rate, reaching up to 49% (5). Identifying the disease severity early is critical in reducing overall mortality.

CURB-65 and pneumonia severity index (PSI) are the most frequently used score systems in ICU for assessing pneumonia severity and predicting mortality. However, existing data suggest that CURB-65 is not a good tool for mortality prediction in pneumonia patients with malignancy (6). The PSI uses more relevant prognostic variables than CURB-65, but cumbersome operation and insufficient timeliness limit its application in emergency settings. Beyond that, researches have shown that these disease-specific scoring systems may be less effective in assessing pneumonia severity in elderly patients compared to younger adults (7, 8). Notably, there is a paucity of prior studies that have developed a predictive model specifically for determining the short-term prognosis of ICU-admitted patients with SCAP.

Therefore, the main purpose of this study was to develop a nomogram for the prediction of short-term death in ICU-admitted SCAP patients.

## 2 Materials and methods

### 2.1 Data source

Two primary databases were employed in the retrospective study: the Medical Information Mart for Intensive Care IV (MIMIC-IV) database and the electronic medical record database of Chongqing General Hospital.

Both the training set and internal validation set data were exclusively obtained from the MIMIC-IV database, a freely accessible and extensive repository (9). It comprises essential care data of 73,181 patients at the Beth Israel Deaconess Medical Center (Boston, USA) from 2008 to 2019. These records cover various details, including demographic characteristics, diagnosis, vital signs, laboratory results, imaging reports, and surgical information (10). For this research, the datasets were selected from the most recent release of MIMIC-IV (version 2.2) which became available in January 2023. The extraction of the included datasets was performed by Yu Zhang, who successfully completed the institution training initiative program course (Record ID: 51169373). As this study utilized an anonymized public database and adhered to review committee agreements, no ethical consent was required.

For the external validation set, 97 patients with SCAP admitted to the ICU at Chongqing General Hospital were retrospectively included. This tertiary teaching hospital in China served as the study site between January 2020 and July 2023. The protocol of this study has been approved by the Ethics Committee of the Chongqing General Hospital (KY S2022-099-01). The informed consent was waived, considering that the current project had no impact on clinical care and all patients were de-identified.

### 2.2 Study population

As per the consensus guidelines established by the Infectious Diseases Society of America/American Thoracic Society (IDSA/ATS) (11), the inclusion criteria for patients were defined as meeting any one of the major criteria (such as requiring mechanical ventilation due to respiratory failure or septic shock necessitating vasopressors) or meeting at least three minor criteria (such as having respiratory rate ≥ 30 breaths/min, PaO2/FiO2 ratio ≤ 250, experiencing multilobar infiltrates, exhibiting confusion or disorientation, having a blood urea nitrogen level ≥ 20 mg/dL, a white blood cell count < 4,000 cells/μL, a platelet count < 100,000 cells/μL; a core temperature < 36°C, or experiencing hypotension requiring intensive fluid resuscitation).

Patients who met the following criteria were excluded from the study: being under 18 years of age, voluntarily discontinuing treatment, being pregnant or breastfeeding, or requiring a surgical operation.

### 2.3 Data collection

In this study, the following general information were gathered: the participants’ age, gender, as well as their vital signs upon their initial admission to the ICU. Existing comorbidities were also considered, such as myocardial infarction (MI), pulmonary heart disease (PHD), congestive heart failure (CHF), hypertension, bronchiectasis, chronic pulmonary disease, cerebrovascular disease (CVD), diabetes, renal disease, severe liver disease, and malignant cancer. The analysis involved examining the results of various laboratory tests, including blood cell count, coagulation assessment, hepatic and renal function, electrolyte levels, and arterial blood gas analysis. Chest radiological images of bronchiectasis, pleural effusion, consolidation, and air bronchogram were assessed. The clinical management of the participants involved determining if they required invasive mechanical ventilation, vasopressors, glucocorticoids, and two or more different antibiotics. Lastly, the mortality within 30 days of ICU admission was evaluated as the primary outcome.

Data collection occurred within the initial 24 h period of admission, opting for the most pathological value in cases laboratory examinations were repeated more than once. Data analysis solely involved the first ICU stay for patients with multiple admissions. At least two researchers re-checked these data for accuracy independently.

### 2.4 Statistical analysis

In this study, R software, version 4.3.1 (R Foundation for Statistical Computing, Vienna, Austria) was utilized for statistical analysis. Patients with missing values and candidate variables with over 20% missing data were excluded from the analysis. The “caret” R package was used to randomly split patients from the MIMIC-IV database into a 70% training set for the development of nomogram and a 30% testing set for independent internal validation. The baseline characteristics of survivors and non-survivors were compared. Categorical variables were described using frequencies (n) and percentages (%) and their comparisons were conducted using X^2^ tests. Continuous variables were presented as median (IQR) and compared using the Wilcoxon rank-sum test. All the statistical tests were two-sided, and *p*-value less than 0.05 was deemed to be statistically significant.

The least absolute shrinkage and selection operator (LASSO) was used to perform regression on the training set, and potential predictors were identified using the R package “glmnet.” A 10-fold cross-validation method was employed to find the lambda.1se in order to determine independent risk factors. Risk factors selected by LASSO method were included in a multiple logistic regression analysis using backward stepwise selection. Multicollinearity was evaluated by variable inflation factors (VIF). The development of predictive model was carried out using the R packages “rms.” Ultimately, a nomogram for predicting 30-day mortality was presented based on the predictive model.

The prediction accuracy was evaluated by using the area under the curve (AUC) of the receiver operating characteristic (ROC) curve. To assess the variance between the predicted value derived from nomogram and the actual value, a calibration curve and the Hosmer–Lemeshow goodness of fit test (HL test) were employed. Additionally, the clinical benefits of the predictive nomogram were evaluated through decision curve analysis (DCA).

## 3 Results

### 3.1 Patient selection

**FIGURE 1:**
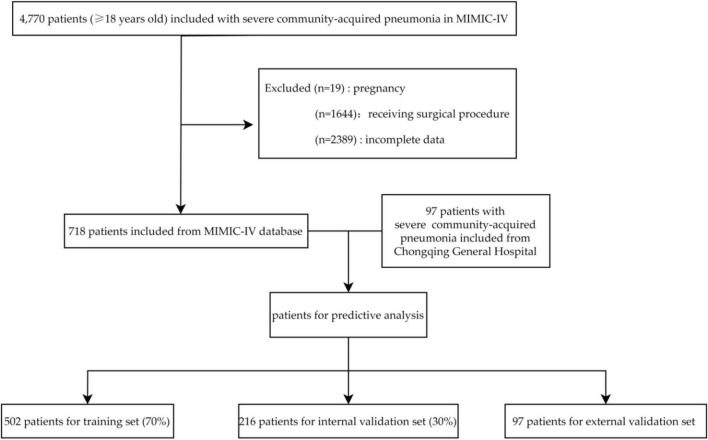
Flowchart of patients selection.

A total of 4,770 SCAP patients were screened in the MIMIC-IV database with reference to the inclusion criteria. Then, 1,644 patients were excluded as they received a surgical procedure. Of the remaining 3,126 patients, 2,408 were excluded due to pregnancy (19) or had incomplete data (2,389). Finally, 718 patients were included in analysis and were arbitrarily divided into two subsets: a training set (*n* = 502, 70%) and an internal validation set (*n* = 216, 30%). Data of 97 patients from Chongqing General Hospital between January 2020 and July 2023 was used for external validation. The patients selection process is shown in Figure 1.

### 3.2 Clinical characteristics

Table 1 provides a detailed comparison of clinical characteristics between patients who survived and those who did not in the training set. The table includes information on age, comorbidities, vital signs, and laboratory results, highlighting significant differences between the two groups. Among the individuals in the training set, 178 (35.5%) were non-survivors and 324 (64.5%) were survivors. We found non-survival group had an older age (70.5 vs. 61.0 years, *p* < 0.001). Moreover, the presence of comorbidities such as malignant cancer (23.0 vs. 8.33%, *p* < 0.001) and severe liver disease (14.0 vs. 7.1%, *p* = 0.018) was significantly associated with mortality. In terms of vital signs, increased heart rate (94 vs. 89 bpm, *p* = 0.003), decreased temperature (37.0 vs. 37.1°C, *p* = 0.005), decreased saturation of peripheral oxygen (96 vs. 97%, *p* = 0.032), and decreased mean arterial pressure (72 vs. 77 mmHg, *p* < 0.001) were associated with mortality. Compared to the survival group, the non-survival group had elevated blood urea nitrogen (41 vs. 27 mg/dL, *p* < 0.001), creatinine (1.60 vs. 1.25 mg/dL, *p* = 0.005), lactate (2.4 vs. 1.7 mmol/L, *p* < 0.001), prolonged prothrombin time (16.4 vs. 14.1 s, *p* < 0.001), and reduced levels of hemoglobin (9.5 vs. 10.6 g/L, *p* < 0.001), platelets (148 vs. 174 × 10^9^/L, *p* = 0.001), lymphocytes (0.84 vs. 1.08 K/μL, *p* < 0.001), albumin (2.8 vs. 3.2 g/dl, *p* < 0.001), pH (7.27 vs. 7.31, *p* = 0.005), and PaO2/FiO2 ratio (107 vs. 129, *p* = 0.033). Additionally, a higher percentage of non-survivors required vasopressor support (66.9 vs. 47.2%, *p* < 0.001).

**TABLE 1:** Clinical characteristics of training set comparing survived vs. non-survived patients.

Variables	Overall (*n* = 502)	Survival (*n* = 324)	Non-survival (*n* = 178)	*p*-value
**Demographics**				
Age, years	65.0 (53.0, 76.0)	61.0 (50.0, 72.0)	70.5 (59.0, 81.8)	<0.001
**Gender (*n*, %)**				0.069
Female	229 (45.6%)	158 (48.8%)	71 (39.9%)	
Male	273 (54.4%)	166 (51.2%)	107 (60.1%)	
Nicotine dependence, yes (*n*, %)	195 (38.8%)	134 (41.4%)	61 (34.3%)	0.143
Alcohol abuse, yes (*n*, %)	81 (16.1%)	59 (18.2%)	22 (12.4%)	0.115
**Comorbidities**				
Hypertension, yes (*n*, %)	287 (57.2%)	183 (56.5%)	104 (58.4%)	0.744
Myocardial infarct, yes (*n*, %)	80 (15.9%)	54 (16.7%)	26 (14.6%)	0.634
Congestive heart failure, yes (*n*, %)	169 (33.7%)	114 (35.2%)	55 (30.9%)	0.382
Pulmonary heart disease, yes (*n*, %)	21 (4.18%)	16 (4.94%)	5 (2.81%)	0.364
Bronchiectasis, yes (*n*, %)	3 (0.60%)	2 (0.62%)	1 (0.56%)	1.000
Chronic pulmonary disease, yes (*n*, %)	84 (16.7%)	60 (18.5%)	24 (13.5%)	0.187
Cerebrovascular disease, yes (*n*, %)	63 (12.5%)	36 (11.1%)	27 (15.2%)	0.241
Diabetes, yes (*n*, %)	141 (28.1%)	86 (26.5%)	55 (30.9%)	0.350
Renal disease, yes (*n*, %)	105 (20.9%)	61 (18.8%)	44 (24.7%)	0.150
Malignant cancer, yes (*n*, %)	68 (13.5%)	27 (8.33%)	41 (23.0%)	<0.001
Severe liver disease, yes (*n*, %)	48 (9.56%)	23 (7.10%)	25 (14.00%)	0.018
**Vital signs**				
Temperature, °C	37.1 (36.7, 37.5)	37.1 (36.8, 37.6)	37.0 (36.5, 37.4)	0.005
Heart rate, bpm	92 (78, 104)	89 (77, 102)	94 (81, 108)	0.003
Respiratory rate, bpm	22 (18, 25)	21 (18, 25)	22 (19, 26)	0.077
SpO2, %	96 (95, 98)	97 (95, 98)	96 (94, 98)	0.032
MAP, mmHg	75 (70, 82)	77 (71, 83)	72 (68, 78)	<0.001
**Laboratory results**				
Hemoglobin, g/L	10.2 (8.6, 11.8)	10.6 (9.1, 12.1)	9.5 (8.0, 11.1)	<0.001
Platelets, K/μL	166 (103, 236)	174 (116, 240)	148 (67, 223)	0.001
WBC, K/μL	14.3 (9.8, 20.2)	13.8 (9.8, 19.8)	15.3 (10.1, 20.8)	0.269
Lymphocytes, K/μL	1.01 (0.60, 1.53)	1.08 (0.67, 1.58)	0.84 (0.44, 1.40)	<0.001
Monocytes, K/μL	0.62 (0.34, 1.00)	0.63 (0.38, 0.99)	0.60 (0.26, 1.03)	0.166
Neutrophils, K/μL	10.20 (6.56, 15.80)	10.00 (6.56, 15.60)	10.70 (6.66, 16.10)	0.682
ALB, g/dL	3.00 (2.60, 3.50)	3.20 (2.70, 3.60)	2.80 (2.30, 3.30)	<0.001
BUN, mg/dL	31.0 (20.0, 50.8)	27.0 (17.0, 44.0)	41.0 (27.0, 58.0)	<0.001
Creatinine, mg/dL	1.40 (0.90, 2.40)	1.25 (0.90, 2.20)	1.60 (1.10, 2.60)	0.005
PT, s	14.7 (13.1, 18.2)	14.1 (12.8, 16.6)	16.4 (13.7, 21.8)	<0.001
Sodium, mEq/L	141 (137, 144)	141 (138, 144)	141 (136, 146)	0.719
Potassium, mEq/L	4.60 (4.10, 5.20)	4.50 (4.10, 5.20)	4.65 (4.12, 5.30)	0.088
Chloride, mEq/L	106 (101, 111)	106 (101, 111)	106 (100, 112)	0.931
pH	7.30 (7.20, 7.37)	7.31 (7.21, 7.38)	7.27 (7.17, 7.36)	0.005
Lactate, mmol/L	2.00 (1.30, 3.48)	1.70 (1.20, 2.80)	2.40 (1.60, 4.50)	<0.001
PaO2/FiO2 ratio	121.0 (80.0, 192.0)	129.0 (82.9, 199.0)	107.0 (73.4, 182.0)	0.033
**Radiographic findings**				
Atelectasis, yes (*n*, %)	158 (31.5%)	102 (31.5%)	56 (31.5%)	1.000
Consolidation, yes (*n*, %)	128 (25.5%)	82 (25.3%)	46 (25.8%)	0.981
Pleural effusion, yes (*n*, %)	257 (51.2%)	169 (52.2%)	88 (49.4%)	0.624
Air bronchogram, yes (*n*, %)	12 (2.39%)	9 (2.78%)	3 (1.69%)	0.552
**Medical management (during the first 24 h after admission)**				
Requirement for vasopressors, yes (*n*, %)	272 (54.2%)	153 (47.2%)	119 (66.9%)	<0.001
Requirement for invasive ventilation, yes (*n*, %)	328 (65.3%)	205 (63.3%)	123 (69.1%)	0.224
Requirement for glucocorticoids, yes (*n*, %)	140 (27.9%)	84 (25.9%)	56 (31.5%)	0.223
Requirement for 2 (or more) antibiotics, yes (*n*, %)	407 (81.1%)	256 (79.0%)	151 (84.8%)	0.141

SpO2, saturation of peripheral oxygen; MAP, mean arterial pressure; WBC, white blood cells; ABL, albumin; BUN, blood urea nitrogen; PT, prothrombin time.

### 3.3 Predictors selection and model development

**FIGURE 2:**
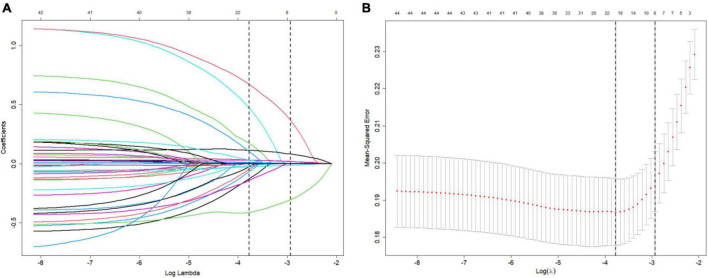
Variable selection using LASSO (The least absolute shrinkage and selection operator) and cross-validation. **(A)** Coefficient trendlines depict the relationship between 44 clinically relevant factors and 30-day mortality. **(B)** Determination of parameter (lambda) selection for deviance in LASSO regression using both the minimum criteria and the 1 standard error criteria.

Figure 2 depicts the process of prediction variable selection using LASSO regression and cross-validation. Panel (a) displays the coefficient trendlines of 44 clinically relevant factors for 30-day mortality, while panel (b) demonstrates the determination of parameter (lambda) selection for deviance in LASSO regression, utilizing both the minimum criteria and the 1 standard error criteria.

Table 2 outlines the results of the multivariable logistic regression analysis conducted on the training set data. The included predictors for predicting 30-day mortality in severe community-acquired pneumonia (SCAP) patients in the intensive care unit (ICU) are age (OR: 1.043, 95% CI: 1.028–1.06), presence of malignant cancer (OR: 2.763, 95% CI: 1.531–5.042), heart rate (OR: 1.019, 95% CI: 1.006–1.033), mean arterial pressure (OR: 0.962, 95% CI: 0.938–0.987), albumin (OR: 0.571, 95% CI: 0.4–0.805), blood urea nitrogen (OR: 1.009, 95% CI: 1.001–1.017), prothrombin time (OR: 1.044, 95% CI: 1.01–1.08), and lactate levels (OR: 1.174, 95% CI: 1.074–1.291). Among these predictive factors, presence of malignant cancer is a categorical variable, while the rest are continuous variables.

**TABLE 2:** Multivariable logistic regression model for predicting 30-day mortality in training set.

Predictive factors	β	OR (95% CI)	*p*-value
Age	0.042	1.043 (1.028–1.060)	<0.001
Malignant cancer	1.016	2.763 (1.531–5.042)	0.001
Heart rate	0.019	1.019 (1.006–1.033)	0.004
MAP	-0.038	0.962 (0.938–0.987)	0.003
ABL	-0.561	0.571 (0.400–0.805)	0.002
BUN	0.009	1.009 (1.001–1.017)	0.034
PT	0.043	1.044 (1.010–1.080)	0.011
Lactate	0.161	1.174 (1.074–1.291)	0.001

MAP, mean arterial pressure; ABL, albumin; BUN, blood urea nitrogen; PT, prothrombin time; OR, odds ratio; CI, confidence interval.

**FIGURE 3:**
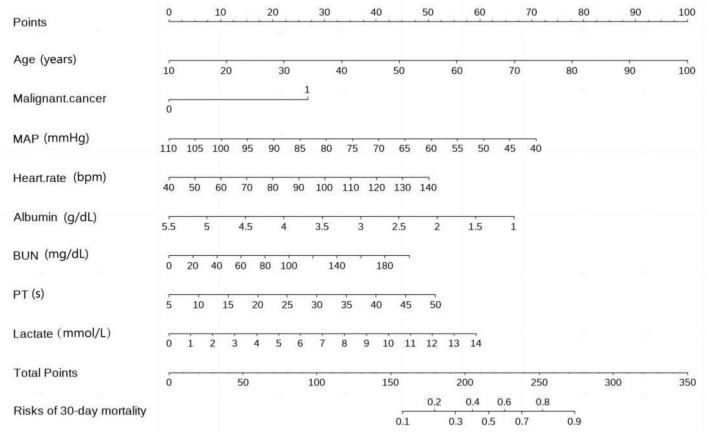
Nomogram for 30-day mortality prediction in ICU-admitted SCAP (severe community-acquired pneumonia) patients.

According to the collinearity check results, the variance inflation factor (VIF) value for all covariates were less than 1.5, suggesting no significant collinearity. Finally, Figure 3 displays a nomogram designed for predicting 30-day mortality in patients with severe community-acquired pneumonia (SCAP) admitted to the intensive care unit (ICU). The nomogram incorporates eight distinct risk factors, including age, presence of malignant cancer (represented as 1 for presence and 0 for absence in the nomogram), heart rate, mean arterial pressure (MAP), albumin (ABL), blood urea nitrogen (BUN), prothrombin time (PT), and lactate levels. Each factor is assigned a corresponding score, the total point was assessed by summing each score from these 8 selected factors. On the bottom of the nomogram, the probabilities of 30-day mortality were predicted in terms of the total scores.

### 3.4 Model performance

**FIGURE 4:**
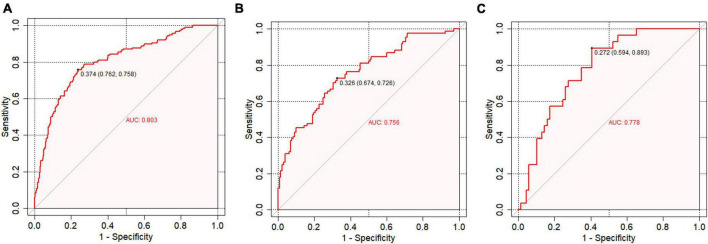
Receiver operating characteristic (ROC) curve analysis. **(A)** ROC curve analysis for the training set; **(B)** ROC curve analysis for the internal testing set; **(C)** ROC curve analysis for the external testing set.

Figure 4 presents the receiver operating characteristic (ROC) curve analysis for the predictive model’s accuracy in the training set (a), internal testing set (b), and external testing set (c). The ROC curve visually illustrates the model’s ability to discriminate between survivors and non-survivors, with the area under the curve (AUC) values providing a quantitative measure of the model’s performance. The ROC curve is employed to evaluate the predictive accuracy. In the training set, the AUC was 0.803 (95% CI: 0.756–0.845). Similarly, in the external validation set, it was 0.778 (95% CI: 0.594–0.893), while in the internal validation set, the AUC was 0.756 (95% CI: 0.693–0.816).

**FIGURE 5:**
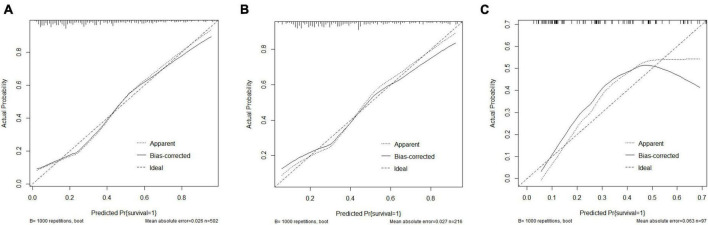
Calibration curve analysis. **(A)** Calibration curve analysis in the training set; **(B)** Calibration curve analysis in the internal testing set; **(C)** Calibration curve analysis in the external testing set.

Figure 5 depicts the calibration curve analysis for the predictive model’s performance in the training set (a), internal testing set (b), and external testing set (c). The calibration curve visually assesses the agreement between the predicted and observed probabilities of 30-day mortality, with the Hosmer–Lemeshow goodness-of-fit test providing a statistical measure of the model’s calibration. The Hosmer–Lemeshow test demonstrated an excellent agreement between prediction and observation (*p* = 0.140 for training set; *p* = 0.161 for internal validation set; *p* = 0.792 for external validation set).

**FIGURE 6:**
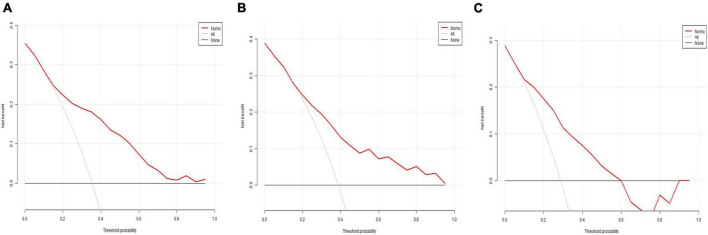
Decision curve analysis (DCA). **(A)** DCA for the training set; **(B)** DCA for the internal testing set; **(C)** DCA for the external testing set.

Figure 6 displays the decision curve analysis (DCA) for the predictive model’s clinical utility in the training set (a), internal testing set (b), and external testing set (c). DCA evaluates the net clinical benefit of the model across a range of threshold probabilities, illustrating its potential practical value in decision-making for predicting 30-day mortality in severe community-acquired pneumonia patients in the ICU.

## 4 Discussion

The prognosis for patients suffering from SCAP is unfavorable, with a mortality rate of 17–49% within 30 days (5). To prevent unnecessary deaths, it is crucial to have an early, efficient, objective, and accurate tool to assess adverse outcomes. Based on two databases, the Medical Information Mart for Intensive Care IV (MIMIC-IV) and the electronic medical record database of Chongqing General Hospital, this study developed and verified a nomogram that can predict 30-day mortality in ICU patients with SCAP using LASSO and logistic regression analyses. The predictive model identified eight variables that were independently statistically significant, including age, presence of malignant cancer, heart rate, MAP, ABL, BUN, PT, and lactate levels. All eight parameters can be easily and simply measured within 24 h of admission. Furthermore, integrating various fundamental clinical characteristics such as age, comorbidities, vital signs, and laboratory tests, the model exhibited excellent predictive performance, broad applicability, and clinical utility in both internal and external validation groups.

Several tools for assessing the severity of community-acquired pneumonia, such as the PSI and CURB-65 have been clinically validated for determining the need for ICU admission and predicting mortality. Factors such as age, presence of malignant cancer, heart rate, MAP, and BUN levels are included in either the PSI score or the CURB-65 score. In addition, this study found raised BUN concentration, prolonged PT, and high lactate levels to be significantly linked to a higher risk of death within 30 days.

Previous studies have indicated a significant age disparity between survivors and non-survivors (12–15). In line with these previous findings, this study has corroborated that the mortality rate of patients with SCAP rises with age. Elderly individuals tend to possess a diminished immune system and decreased somatic function (16). Moreover, increasing age serves as a contributing factor to the susceptibility of multidrug-resistant pathogens, heightening the potential for adverse outcomes (17). Notably, the presence of malignant cancer emerges as the most significant risk factor, exhibiting an odds ratio of 2.763 in the predictive model, which aligns with findings from PSI. Data from recent studies support that malignancy is a predictor of adverse outcomes in adult patients with pneumonia (18, 19). This association may be attributed to the immunosuppressive state caused by cancer, promoting easier pathogens infiltration, as well as the risks associated with intensive chemotherapy that leads to potentially life-threatening complications.

It is generally believed that the pathogenesis of SCAP involves multiple factors such as pathogens and inflammatory cascades that can lead to multiple organ dysfunction and ultimately death. SCAP patients are prone to complications, including sepsis, acute kidney injury (AKI) and coagulation abnormalities, etc. Among these, septic shock is the most lethal complication of SCAP (20). Clinical features of tachycardia and low blood pressure indicate circulatory failure, they are also pivotal variables in predicting unfavorable outcomes in SCAP patients (21, 22). Consistent with this conclusion, we observed that the non-survival group had a faster heart rate and lower mean arterial pressure than the survival group. Meanwhile, higher lactate suggests insufficient tissue perfusion, circulatory disorder, increased vascular permeability, and damaged blood vessel walls (23). It also is reported to be associated with higher hospital mortality in SCAP patients (24, 25).

Low serum albumin at admission was considered as an independent risk parameter for predicting mortality in CAP patients (26). In a study exploring CAP patients, Ma et al. (27) found that lower levels of albumin at admission were indicative of higher short-term mortality. There are two reasons that contribute to this relationship. Firstly, low serum albumin is frequently associated with inadequate nutrition and poorer recovery (28). Secondly, the albumin molecule plays a critical role in immunomodulatory effects and regulates immune responses against invasive pathogens (29).

Results of published studies have demonstrated that CAP can increase the risk of developing AKI. Characterized by raised BUN concentration, AKI was independently associated with the short-term mortality of CAP patients (30, 31). Earlier studies indicated that individuals who did not survive CAP had elevated BUN levels (32, 33). Consistent with previous studies on SCAP, this investigation revealed that the BUN level remained a significant predictive indicator of mortality, as demonstrated by multivariate analysis.

It is universally acknowledged that PT reflects the function of the extrinsic coagulation pathways. Prolonged PT indicates consumption of coagulation factors caused by activation of coagulation. Increasing evidence shows that coagulation disorders are involved in the progress of severe pneumonia (34, 35). Among the SCAP patients included in this study, non-survivors had a longer PT, which confirmed the findings of the previous studies.

Huang et al. established a nomogram to assess the risks of hospital mortality among 611 patients who had both SCAP and chronic obstructive pulmonary disease (COPD). This nomogram included various factors such as chronic renal diseases, diabetes, systolic blood pressure, interleukin-6 (IL-6), fibrinogen, and BUN (AUC: 0.840, 95% CI: 0.809–0.872) (36). In a recent study by Song et al. (37) a similar nomogram was developed to predict the 28-day mortality among elderly SCAP patients. This nomogram included factors like age, Glasgow score, platelet count, and BUN (AUC: 0.713, 95% CI: 0.646–0.767) (37). Although these two models showed sound performance in discrimination and calibration, restrictions on age and comorbidities of enrolled patients limit their range of application. Furthermore, score systems, like Glasgow Coma Scale, included in a model impact its promptness and evaluation efficiency. Compared with previous studies, the present study has several advantages. First, this study focused exclusively on patients in ICU settings and is suitable for all severe community-acquired pneumonia caused by infectious diseases. Fewer restrictions on enrolled adult SCAP patients enable broader applicability of the prediction model, regardless of specific comorbidities or specific age groups. Then, this study included a wider range of independent risk factors, encompassing comorbidities, indicators of shock, renal function index, and coagulation function index. All these factors can be obtained promptly and directly after admission, which ensures the model’s simplicity and timeliness. Meanwhile, the study identified eight risk factors associated with 30-day mortality in SCAP patients. While age, presence of malignant cancer, and lactate levels have been reported by most studies, this study not only confirmed these findings but also analyzed the impact of serum albumin, BUN, PT, heart rate and MAP on short-term prognosis, which allows a more quantitative prediction compared to usual bedside evaluation. In addition, with excellent performance in discrimination and calibration indicated by AUC and calibration curve, the nomogram has great potential in clinical promotion, especially in primary medical institutions or underdeveloped regions. Third, the study enrolled patients from two hospitals in different countries, and the outcomes observed in the training set were consistent with those in the validation set, thereby enhancing the generalizability of the nomogram.

Despite the strengths mentioned earlier, this study has unavoidable limitations. Firstly, the lack of a standardized description for “multilobar infiltration” in chest radiological reports led to the exclusion of some eligible severe community-acquired pneumonia (SCAP) patients from the MIMIC-IV database. This non-uniformity in reporting may have impacted the comprehensiveness of our patient selection. Secondly, the retrospective nature of the study, including patients from a single tertiary hospital in China for external validation, poses limitations. The relatively small sample size, despite internal and external validation efforts to assess the nomogram’s robustness, may constrain the generalizability of our findings. External validation in larger and more diverse SCAP populations is essential to ascertain the nomogram’s applicability to other hospitals in China. Thirdly, the exclusion of patients with incomplete data might introduce selection bias, underscoring the need for further prospective, multi-center studies to ensure both generalizability and complete data collection. Additionally, the deliberate exclusion of post-operative patients, aimed at mitigating confounding factors, acknowledges certain limitations to the external validity and breadth of the predictive model. Recognizing these constraints, future studies should address the unique challenges posed by SCAP in post-operative settings to offer a more holistic perspective on mortality risk assessment in this specific population. Furthermore, the analysis of risk factors did not encompass all potential predictors influencing mortality in ICU-admitted SCAP patients. Notably, inflammatory indicators and specific pathogenic bacteria species were not thoroughly evaluated due to missing data in the MIMIC-IV database. Consequently, the nomogram may require updating or recalibrating in subsequent studies.

## 5 Conclusion

In conclusion, this study developed a rapid, cost-effective nomogram for predicting 30-day mortality in ICU-admitted patients with severe community-acquired pneumonia (SCAP). The nomogram incorporates eight independent risk factors, namely age, presence of malignant cancer, heart rate, mean arterial pressure, albumin, blood urea nitrogen, prothrombin time, and lactate levels. Validation showed strong potential for discrimination, calibration, and clinical utility of the predictive model. Despite this, prospective cohort studies and further validation in diverse populations are warranted to refine and enhance the generalizability of the nomogram.

## Data availability statement

The datasets generated and analyzed during the current study are available from corresponding authors upon reasonable request.

## Ethics statement

The studies involving humans were approved by the Ethics Committee of Chongqing General Hospital. The studies were conducted in accordance with the local legislation and institutional requirements. The ethics committee/institutional review board waived the requirement of written informed consent for participation from the participants or the participants’ legal guardians/next of kin because the current project had no impact on clinical care and all patients were de-identified.

## Author contributions

YZ: Conceptualization, Data curation, Formal analysis, Methodology, Software, Writing – original draft. YP: Data curation, Writing – original draft. WZ: Funding acquisition, Investigation, Project administration, Supervision, Writing – review & editing. WD: Funding acquisition, Investigation, Project administration, Supervision, Writing – review & editing.
